# Popularity and Friendships and Their Relationship to Physical Activity Before and After Transition to a Higher School Grade

**DOI:** 10.3390/ijerph16152782

**Published:** 2019-08-03

**Authors:** Kenda C. Swanson, Alberto Nettel-Aguirre, Gavin R. McCormack

**Affiliations:** 1 Department of Community Health Sciences, Cumming School of Medicine, University of Calgary, Calgary, AB T2N 4N1, Canada; 2 Department of Paediatrics, Cumming School of Medicine, University of Calgary, Calgary, AB T2N 4N1, Canada

**Keywords:** exercise, child, school, social support, social network, friendship, peers, physical activity

## Abstract

*Background* This study investigated the relationships between children’s friendship ties and their physical activity (PA) both before and after their transition to a new school year. *Methods* In 2011–2012, children in grades 5–8 attending a Canadian urban middle-school completed web-based health and friendship surveys two times before (“pre-transition”) and three times after (“post-transition”) they moved up in school grade. Cross-sectional associations between an average daily frequency of ≥60 min/day of moderate-to-vigorous intensity PA (MVPA) and characteristics of children’s friendships were estimated for pre-transition (*n* = 191) and post-transition (*n* = 255) data. Sociodemographic-adjusted linear regression (β) estimated associations between a child’s MVPA and friendship characteristics. *Results* We found positive associations between a child’s MVPA and the average MVPA of their friends at post-transition only (β = 0.61, 95% CI 0.10 to 1.13) and the number of sent friendships at pre-transition (β = 0.03, 95% CI 0.01 to 0.05) and post-transition (β = 0.02, 95% CI 0.01 to 0.04). A statistically significant interaction between popularity and friends’ average PA at pre-transition was also found. *Conclusions* The PA of friends and the number of school friends that a child identified are positively associated with MVPA. The estimated associations between MVPA and aspects of children’s friendships are similar for boys and girls.

## 1. Introduction

Regular participation in physical activity may lead to increased opportunities for interaction and engagement and has positive implications for bonding and friendship formation among children [1]. Moreover, physical activity habits formed during childhood may be continually reinforced throughout adolescence and be carried into adulthood [2]. Thus, promoting regular participation in physical activity from an early age is important [3,4].

There is continued interest in how the social environment influences children’s health behaviours [5,6,7]. Evidence suggests that aspects of peer relationships are related to children’s physical activity; specifically, the number of sent friends (total number of outgoing friendship nominations, regardless of whether they were reciprocated) has been linked to physical activity [5,8,9], and friends tend to engage in similar amounts of physical activity [10,11]. Longitudinal studies employing a social network analysis have found that over time, friends’ activity levels become even more similar through the processes of peer influence (children emulating their friends’ behaviours) [12] and selection, whereby children may be more likely to select friends who already share similar physical activity behaviours [12]. Previous studies have also found evidence that popular children are more likely to be active and play sports than those with fewer friendship ties to other children [11,13]. Interventions that capitalize on the potential influence of children’s social networks on their physical activity may result in improved and sustained behaviour change.

Notably, boys tend to be more active than girls, and some evidence suggests that the decline in physical activity that occurs during adolescence is steeper for girls [2,14,15]. Boys and girls may be motivated to participate in physical activity for different reasons, which may explain the observed differences in their activity patterns. For example, girls tend to be drawn to the social aspects of activities, while boys are interested in the competition involved in sports [16]. Despite equivocal findings, several studies have found that the association between friendship and physical activity exists among boys only [17,18], or that it is stronger for boys than it is for girls [12,19]. A better understanding about whether there are sex differences in the relationship between aspects of children’s friendships and their physical activity could inform future physical activity interventions and may result in the development and implementation of specific strategies incorporating social networks to improve physical activity in boys and girls.

Transitioning to a higher school grade is an important time for children. Aspects of children’s mental health and wellbeing are commonly assessed health outcomes in the school transition literature (e.g., feelings of stress and well-being) [20]. However, only a small quantity of published studies have investigated the way in which school grade transitions are related to aspects of children’s *physical* health, such as their physical activity [20,21,22]. Garcia and colleagues [22] identified positive associations between physical activity beliefs and physical activity both pre- and post-transition from elementary to junior high school and found that pre-transition physical activity was predictive of post-transition physical activity. Jago et al. [23] found that children’s physical activity declined over the transition from primary to secondary school, and an increase in the number of friends over the transition was associated with higher levels of physical activity. This evidence suggests that transitioning to a higher school grade may influence children’s friendships and their physical activity [22,23]. Moreover, it is possible that school grade transitions disrupt school-based social and friendship networks that subsequently impact physical activity among children.

Studies have yet to investigate the associations between the characteristics of children’s peer friendships and their physical activity before and after they transition school grade within the same school. Focusing on children undergoing a grade transition within the same school is important as it controls for the potential confounding that could result from exposure to a new school environment during a school transition (any observed changes in physical activity might be the result of a change in school physical environment and the independent effect of a change friendship network on physical activity before and after school transition might be difficult to isolate). Thus, we sought to investigate whether measures capturing different types of friendships among children, including friends’ average physical activity, popularity (number of incoming friendship nominations), and sent friendships, were associated with a child’s physical activity, pre- and post-transition to a higher school grade within the same school. 

Given previous evidence suggesting that aspects of children’s peer friendships, including friends’ average physical activity and popularity, are positively related to an individual’s own physical activity [10,24], we expected to find positive associations between aspects of children’s peer friendship ties and their physical activity pre- and post-transition to a higher school grade within the same school. The findings of this study provide insight as to which characteristics of children’s peer friendships are related to individual physical activity (if any), clarify the direction and magnitude of these associations, and provide insight as to whether the associations are different between boys and girls. Understanding how children’s peer friendships can influence physical activity may contribute to the development of school-based interventions that aim to maintain and/or improve children’s physically activity, especially after school grade transitions. 

## 2. Materials and Methods 

### 2.1. Study and Sample Design

In 2011, consent forms were sent home to all the parents of *n* = 294 children in grades 5 (starting ages 10–11 years), 6 (starting ages 11–12 years) and 7 (starting ages 12–13 years) attending an urban public middle school in Calgary, Alberta, Canada. The school was selected because our research team had been undertaking observational data collection in the elementary school from which children transitioned prior to the data collected for this study. All the children included in this study returned completed consent forms, signed by their parents/guardians. The school was in a neighbourhood with a median household income of $65,220 in 2011—lower than the median income for Calgary ($81,256) [25]. The University of Calgary Conjoint Health Research Ethics Board granted ethics approval for this study (REB#6771).

Data were collected on five occasions comprising before (pre-transition 1: March 2011, pre-transition 2: May 2011) and after (post-transition 3: October 2011, post-transition 4: February 2012, post-transition 5: April 2012) children transitioned to a higher school grade. The school year in Calgary is from September to June. Restrictions in relation to school curriculum programing and scheduling, permission from the Calgary Board of Education, and our need to collect data at multiple time points during the school year determined when and how many times we could administer the surveys. Of the *n* = 294 parents/children approached, *n* = 256 (87%) children participated in the study in year one, of which n = 191 provided complete data (response rate = 65%). Following the transition to a new school year, including new student enrollments, *n* = 399 children in grades 6–8 were sent home with consent forms, of which *n* = 255 provided complete data (response rate = 64%). Among all the students who participated in the study, *n* = 130 provided complete data both before and after the grade transition.

At each data collection period, the children completed two web-based questionnaires which were administered together during school hours. Members of the research team oversaw the data collection and were available to assist students with interpreting any questions or handling the technology. Among other characteristics, the first questionnaire captured physical activity and sociodemographic variables, including family affluence. The second questionnaire involved a survey of peer friendships, which captured school-based friend nominations of whom in the class or grade a child was friends with. In surveys of peer friendships, children in grade 5 and 6 were instructed to nominate any peers within their own class as friends, whereas children in grades 7 and 8 were instructed to nominate any peers within their own grade. This difference reflected the fact that grade 5 and 6 students remained in the same class throughout the school day, whilst grades 7 and 8 students changed classrooms throughout the day. The two questionnaires took approximately 35 min in total to complete. Notably, when students transitioned to a new grade, they did not share the same classroom with all the same students from the previous year. Therefore, this transition disrupted the friendship network and provided opportunities for new networks to form. Thus, we assumed that as children transitioned to new classrooms following the transition in grade, their school-based friendship ties would be impacted and that this disruption could be associated with their physical activity.

### 2.2. Measures 

#### 2.2.1. Physical Activity

At the time this study was conducted, Canadian guidelines recommended that children and adolescents aged 5 to 17 years participate in at least 60 min of moderate-to-vigorous intensity physical activity daily [26]. Two items captured the children’s frequency of achieving at least 60 min of moderate-to-vigorous intensity physical activity per day during the “last” and “typical” week, respectively. Specifically, children were asked to identify how many days (on a scale from 0–7) they were “physically active for at least 60 min per day.” Specifically, they were asked to report “any activity where you get out of breath some of the time, like running, biking, skiing, sports and playing tag,” over the past 7 days, and in a typical week [27]. The two items did not capture physical activity undertaken as part of the physical education class but did capture activity undertaken during recess and lunch time. Responses to the two items were highly correlated (r = 0.77) and combined (i.e., averaged) into a single physical activity index (i.e., scores from 0 to 7), the same as previous studies [10,27,28]. Others have found moderate correlations (r = 0.40) between this physical activity index and accelerometer-measured physical activity [27,28]. Data collected prior to the grade transition were averaged (pre-transition 1, pre-transition 2), and data collected after the transition to a new school grade were averaged (post-transition 3, post-transition 4, post-transition 5), each providing a habitual measure of physical activity over the school year [29,30].

#### 2.2.2. Sociodemographic Characteristics

Items captured sex (boy = 0, girl = 1), age in years (continuous), residential mobility (did not move in the past 12 months = 0, or moved ≥1 times in the past 12 months = 1), years residing in Canada (<2 years = 0, 2–5 years =1, or >5 years = 2), and family structure (i.e., living with both parents in the same house = 0, or any other living arrangement = 1). Household socioeconomic status was estimated using the Family Affluence Scale (FAS) [31]. The FAS captures family vehicle ownership (0, 1, or ≥2 vehicles), computer ownership (0, 1, 2, or >2 computers/laptops), holiday travel over the past 12 months (0, 1, 2, or >2 times in the past 12 months), and the child having their own, unshared bedroom (no = 0 or yes = 1) [31,32]. The responses to the FAS items were summed and the total score was categorized into low (FAS < 6), medium (FAS 6–7), and high (FAS ≥ 8) to reflect groupings that have been used in other studies investigating the relationships between socioeconomic status and obesity-risk behaviour in children [10,32]. The FAS has been applied in previous research investigating children’s socioeconomic status, health and wellbeing [31,33,34] including physical activity [10,32,35], and is correlated with parents’ occupational social status [36]. The FAS has a strong agreement when validated against parents’ responses to the same items [33].

#### 2.2.3. Friendship Characteristics

Children were asked to nominate “who are you friends with?” from a complete list of children either in their class (for grades 5 and 6) or grade (for grades 7 and 8). The difference in class lists (by class or grade) reflected the difference in the structure of the classes corresponding to grade, whereby grade 5 and 6 spend most their school day in one class, and grades 7 and 8 move between different classes during the school day. There was no limit on the number of friends a child could select. Based on the list of child-identified friends, social network analysis software (UCINET 6 (Analytic Technologies: Harvard, MA, USA) [37]) and SPSS version 21.0 (IBM Corp.: Armonk, NY, USA) were used to estimate three variables that captured the characteristics of children’s friendships that may relate to their physical activity: (1) friends’ average physical activity; (2) incoming friendships (i.e., popularity), and; (3) sent friendship nominations. 

In SPSS, friends’ average physical activity of each child was estimated by averaging the physical activity levels of a child’s sent and incoming friendship nominations. Friendship characteristics were estimated in UCINET. We included the number of incoming friendships to examine the relationship between popularity among children and individual physical activity participation [7]. Popularity estimated the number of *incoming* friend nominations a child received, i.e., it is the conceptual definition of incoming friendship nominations. Sent friendships estimated the number of *sent* friendship nominations. We included these measures to capture different types of friendship relations among peers, as previous evidence suggested that the relationship between peer friendships and activity patterns among children is complex and may be dependent upon the way in which “friendship” is defined [7,8,9,10,11,12]. Friendship characteristics were averaged into two scores, pre-transition (comprising pre-transition 1 and pre-transition 2 surveys) and post-transition (comprising post-transition 3, post-transition 4 and post-transition 5 surveys). 

### 2.3. Statistical Analyses

Descriptive statistics, including percentages for the categorical variables (i.e., sex, residential mobility, years residing in Canada, family structure, and family affluence) and average, median, and standard deviations for numerical variables (age in years, physical activity index, friends’ average physical activity, popularity, and sent friendships) were estimated for pre- and post-transition data. 

For the inferential analysis, we averaged the physical activity index and each of the three friendship characteristic variables collected at pre-transition and separately for those collected post-transition. “Complete data” implies that individuals had no missing values for any variables of interest following the list-wise deletion of incomplete cases. If an average value could not be estimated due to missing physical activity data in one of the time points, the case was assigned the average of the values that were available and this was considered the imputed “average” value. For instance, if a case was missing pre-transition 1 physical activity data, then their pre-transition 2 physical activity data were used in the analysis. If a case was missing data for all time periods, then that case had completely missing data and was excluded from the analysis. For pre-transition surveys at pre-transition 1, there were 191 children with complete data (no missing) and at pre-transition 2, there were 188 children (3 were missing physical activity data, which was imputed from the pre-transition 1 value). For post-transition surveys at post-transition 3, there were *n* = 252 (3 missing), for post-transition 4 *n* = 249 (6 missing), and for post-transition 5 *n* = 246 (9 missing) children. Similarly, as with pre-transition data, post-transition missing physical activity values were imputed from the other periods (either post-3, post-4, or post-5) where data were available. 

Children who did not provide responses to the questionnaire used to collect peer friendship information (no nominated friends) were excluded from the study. We estimated intra-class correlations for each of the physical activity index and friendship variables across the multiple measures before and after the grade transition to indicate how strongly the data comprising the variables were correlated with one another at the different time points—to assess consistency in behaviours captured before and after the transition. To compare the averages of physical activity, friends’ average physical activity, popularity, and sent friendships for individuals who provided complete data at both pre-transition (pre-transition 1 and pre-transition 2) and post-transition (post-transition 3, post-transition 4, post-transition 5), paired dependent t-tests were performed (*n* = 130).

We undertook a separate cross-sectional analysis with each of the pre-transition and post-transition data. Multivariable linear regression estimated the unstandardized beta coefficients (β) and 95% confidence intervals (95% CI) for the association between the physical activity index and the friendship characteristics (friends’ average physical activity, popularity, and sent friendships), adjusting for sociodemographic variables (sex, age in years, residential mobility, years residing in Canada, family structure, and family affluence). Based on evidence indicating potential effect modification by sex [7,11,12,38], we entered interaction terms between sex and the three friendship characteristic variables into the regression model. As an additional exploratory step, we also included interaction terms between popularity and friends’ average physical activity, and sent friendships and friends’ average physical activity. We used backward stepwise elimination (Likelihood ratio) on the interaction terms to remove all those that were not statistically significant (*p* > 0.05). R-square was estimated for all the regression models. Analyses were undertaken using SPSS (version 24). Normal P-P plots of the residuals, partial regression plots of each of the independent versus dependent variables, and scatterplots of the residuals versus dependent variables were visually inspected to assess for violation of linear regression assumptions. All the statistical analyses were performed using a significance level (α) of 0.05. 

## 3. Results

### 3.1. Descriptive Statistics

In total, of the 294 children eligible to participate in the study at the first data survey period, 65% (*n* = 191) provided complete data, meaning that they provided responses to all the questions that were examined in the analyses at the first data collection period. The pre-transition and post-transition sample characteristics were similar, including for the children who provided “complete” data at both time points (Table 1). Overall, the two samples included a higher proportion of boys than girls (pre: 52.4%, post: 51.4%, both pre-transition and post-transition: 54.6%), non-movers in the last year (pre:69.6%, post: 72.5%, both pre and post: 68.5%), residents of Canada for >5 years (pre: 82.7%, post: 86.7%, both pre-and post: 85.4%), children living with two parents in the same home (pre: 72.8%, post: 68.6%, both pre-and post: 72.3%), and children from medium (pre: 41.9%, post: 40.0%, both pre and post: 39.2%) and high affluent families (pre: 35.1%, post: 36.9%, both pre and post: 38.5%). 

Intra-class correlations (ICC) for physical activity measured before the grade transition (pre-transition 1, pre-transition 2; ICC = 0.70) and after the grade transition (post-transition 3, post-transition 4, post-transition 5; ICC = 0.59) were moderate. ICCs ranged from moderate to high prior to and following the grade transition for friends’ average physical activity (ICC = 0.72 and ICC = 0.55), popularity (ICC = 0.94 and ICC = 0.88) and sent friendships (ICC = 0.87 and ICC = 0.77), respectively. There was no statistically significant difference in physical activity before (average = 4.92, SD = 1.63 days/week) versus after the grade transition (average = 4.75, SD = 1.49 days/week) (Table 2). Pre-transition physical activity levels between boys and girls were not statistically significantly different from one another (boys: average = 5.03, SD = 1.66 days/week, and girls: average = 4.79, SD = 1.60 days/week), however, boys’ post-transition physical activity (average = 4.97, SD = 1.41 days/week) was significantly (*p* < 0.05) higher than girls’ (average = 4.52, SD = 1.54 day/week).

In terms of friendship, sent and incoming friendship nominations increased pre- to post-transition to a higher school grade (*p* < 0.05); however, friends’ average physical activity significantly decreased from before to after the grade transition (Table 2). Pre-transition (*n* = 191) children had an average number of incoming friends (i.e., popularity) of 18.55 (SD = 13.05), and post-transition (*n* = 255) children received an average number of 20.10 (SD = 10.13) friendship nominations. Pre-transition average sent friendships was 18.60 (SD = 17.41), and 20.10 (SD = 15.99) post-transition to a higher school grade. On average, friends’ average physical activity was 5.17 days/week (SD = 0.51 days/week) pre-transition to a higher school grade, and after the school grade transition, friends’ average physical activity was 4.96 days/week (SD = 0.13 days/week). Among the sub-group of children who provided data at both pre- and post-transition (*n* = 130), there were no changes in physical activity (Table 2). Most children in this study nominated friends of the same sex (pre-transition = 65.6% and post-transition = 78.6%). 

### 3.2. Associations between Friendship Characteristics and Physical Activity 

Before the grade transition, physical activity was positively associated with sent friendship nominations (β = 0.03, 95% CI 0.01 to 0.05, *p* < 0.05), adjusting for all other characteristics. We also found that age was negatively associated with individual physical activity at pre-transition (β = −0.40, 95% CI −0.68 to −0.13, *p* < 0.05), adjusting for all other characteristics. The fully-adjusted main effects only model explained 25% of the total variability in pre-transition physical activity (Table 3). No interaction was found between sex and the friendship characteristics at pre-transition. Notably, at pre-transition, we found a positive interaction between popularity and friends’ average physical activity (β = 0.06, 95% CI 0.01 to 0.10, *p* < 0.05). 

For post school-grade transition, physical activity was positively associated with friends’ average physical activity (β = 0.61, 95% CI 0.10 to 1.13, *p* < 0.05) and sent friendships (β = 0.02, 95% CI 0.01 to 0.04, *p* < 0.05) (Table 3). No other demographic variables were associated with physical activity at post-transition. The fully-adjusted main effects only model explained 20% of total variability in physical activity at post-transition (Table 3). Notably, sex also did not modify the associations between the friendship characteristics and individual physical activity, nor did we find any statistically significant interactions between popularity or sent friendships and friends’ average physical activity at post-transition. Unadjusted and adjusted regression analysis was performed among the sub-group of children who provided data at both pre- and post-transition (*n* = 133), and no statistically significant associations between physical activity and any of the demographic or friendship characteristics were found. 

## 4. Discussion

The findings of our study suggest that aspects of children’s peer social ties are associated with their physical activity. Consistent with other studies, average physical activity levels of friends’ [6,10,24] and the number of nominated friends [13,24] were positively associated with a child’s physical activity. It may be that children find physical activity a socially-desirable quality, and thus imitate the behaviour of their active peers, increasing their own involvement [39]; or perhaps, they participate in similar activity levels as their friends in everyday maintenance of their friendships. Previous studies have also identified positive associations between a child’s sent friendships and individual physical activity [13,24]. It is possible that children who perceive themselves as friendly with their peers are busier and more active in their lives than those who are less connected [5,40]. Moreover, appealing to social cognitive theory, children may be trying to “fit in” and impress perceived friends by demonstrating willingness to try and adopt activities (i.e., enrolling in sports and clubs) that facilitate interactions with their peers in order to build and strengthen social connections [5]. An active social life may correspond with a more physically active lifestyle when children’s day-to-day activities with their friends involve outings such as sports, games and playdates with one another [41]. 

Contrary to some studies [11,13], we did not find associations between popularity and a child’s physical activity, however, these studies [11,13] had different outcome measures (e.g., overweight/obesity, high-calorie food consumption), and different methodologies, including the instruments used to capture peer friendships (e.g., best friends, close friends). A novel finding of our study was the positive association between individual physical activity and the interaction between popularity and the average physical activity of a child’s friends at pre-transition. This suggests that individuals who are more popular and have physically active friends are more physically active themselves. From an intervention planning perspective, popular, physically active children might play important roles in facilitating physical activity in those who are less popular. Notably, similar to other studies of children aged 10 to 14 years, we found that most children reported same-sex ties [11,16]. 

Previous investigation of the relations between children’s friendships and their physical activity have found mixed results for boys and girls [6,7]. We did not find that sex modified the relationships between aspects of their friendships and their physical activity. Indeed, research into the correlates of playing sports suggests that boys and girls are different in their motivation to participate [16,42,43], and that physical activity interventions applied at school may influence the behaviours of boys and girls differently [43]. Future studies should continue to explore the possibility of differences in children’s friendships and their physical activity behaviours according to sex before interventions to increase physical activity are implemented.

Our study has several limitations. Grades 5 and 6 students reported friendships within their classrooms, while grade 7 and 8 students reported friendships within their grades. It is possible that students had friends in other classrooms or grades that may or may not have influenced their physical activity. Friendships outside of the classroom (grade) and school were not captured. It is possible that our results were distorted by the exclusion of out-of-school friendships, especially if these relationships were very close [39] and were associated with out-of-school physical activity (which our measure captured). Moreover, friends’ physical activity for each child was estimated by averaging the physical activity levels of a child’s sent and incoming friendship nominations, which may be biased due to missing self-reported physical activity data and the fact that not all friends participated in the friendship surveys and were therefore excluded from the analysis. We used an established self-report physical activity instrument [27] that captured children’s activity, nevertheless, self-reported physical activity estimates are vulnerable to recall errors and bias. Furthermore, this measure may not have been sensitive enough to detect small shifts in physical activity as it captured the frequency of achieving the recommended levels of physical activity (at least 60 min/day). Future studies should collect and follow-up on physical activity-specific social ties (e.g., friends from school vs. sports teams and clubs vs. neighbourhood) to better understand the types of friendships that may be associated with children’s activity. Friendship surveys may be supplemented by asking children to elaborate on the way in which their friends (friend groups) encourage them to be active. Context-specific physical activity could be assessed by modifying the study and or questionnaire design to inquire about the type (e.g., group or individual activity, organized or unorganized activity), the presence of peers, and the intensity and location of the physical activities undertaken. 

## 5. Conclusions

The PA of friends and the number of school friends that a child identifies are positively associated with days achieving at least 60-min of MVPA. The estimated associations between MVPA and aspects of children’s friendships were similar for boys and girls. Developing and implementing school-based interventions that take advantage of classroom and or grade friendship ties and networks could increase the number of children achieving the recommended levels of physical activity. 

## Figures and Tables

**Table 1:** Sociodemographic backgrounds of children before, after and across the school grade transition.

Sociodemographic Variables	Pre-Transition Sample (*n* = 191)	Post-Transition Sample *(n* = 255)
**Sex (%)**		
Boys	52.4	51.4
Girls	47.6	48.6
**Age in years (mean, SD, (median))**	11.8, 0.9 (12.0)	12.1, 0.9 (12.0)
**Residential mobility (%)**		
Non-mover last year	69.6	72.5
Mover last year	30.4	27.5
**Years residing in Canada**		
<2 years	6.3	4.3
2–5 years	11.0	9.0
>5 years	82.7	86.7
**Family Structure (%)**		
Two parents in the same house	72.8	68.6
One parent or other living arrangement (step-parent)	27.2	31.4
**Family affluence (%)**		
Low (<6)	23.0	23.1
Medium (6–7)	41.9	40.0
High (≥8)	35.1	36.9

**Table 2:** Descriptive statistics for physical activity and friends’ characteristics of children with complete data.

**Independent *t*-Test**	**Pre-Transition ^1^, *n* = 191**	**Post-Transition ^2^, *n* = 255**
	**Mean, SD (Median)**	**Mean, SD (Median)**
Physical activity index	4.92, 1.63 (5.25)	4.75, 1.49 (5.00)
Friends’ average physical activity *	5.17, 0.51 (5.25)	4.96, 0.40 (5.00)
Incoming friendships *	18.55, 13.05 (14.00)	20.10, 10.13 (19.00)
Sent friendships *	18.60, 17.41 (11.00)	20.10, 15.99 (14.33)
**Dependent *t*-Test**	**Pre-Transition ^1^, *n* = 130**	**Post-Transition ^2^, *n* = 130**
	**Mean, SD (Median)**	**Mean, SD (Median)**
Physical activity index	4.87, 1.68 (5.00)	4.90, 1.48 (5.17)
Friends’ average physical activity *	5.16, 0.51 (5.20)	5.00, 0.42 (5.04)
Incoming friendships *	17.06, 12.55 (11.50)	19.75, 10.24 (17.17)
Sent friendships *	16.00, 15.04 (9.50)	18.72, 14.00 (13.33)

*Note*. Higher physical activity index score = more days of physical activity per week; ^1^ Pre-transition consists of data collected at pre-1 and pre-2; ^2^ Post-transition consists of data collected at post-3, post-4, and post-5; * *p* < 0.05 (two-tailed) based on dependent or independent *t*-test.

**Table 3:** Multivariable linear regression estimates (β; 95% CIs) for associations between physical activity, friendship characteristics, and sociodemographic characteristics.

Characteristics	Pre-Transition Model (*n* = 191)	Post-Transition Model (*n* = 255)
	Fully-Adjusted Model	Fully-Adjusted Model
	β (95% CI)	β (95% CI)
**Sex**		
Boys	Ref	Ref
Girls	−0.04 (−0.48, 0.39)	−0.24 (−0.59, 0.11)
**Age in years**	−0.40 (−0.68, −0.13) *	−0.15 (−0.36, 0.05)
**Residential mobility**		
Non-mover last year	Ref	Ref
Mover last year	−0.36 (−0.88, 0.16)	0.19 (−0.21, 0.59)
**Years residing in Canada**		
<2 years	Ref	Ref
2–5 years	0.86 (−0.27, 1.99)	0.45 (−0.57, 1.48)
>5 years	0.64 (−0.36, 1.65)	0.80 (−0.14, 1.73)
**Family structure**		
Two parents in the same house	Ref	Ref
One parent or other arrangement	−0.05 (−0.88, 0.16)	−0.20 (−0.59, 0.19)
**Family affluence scale**		
Low (<6)	Ref	Ref
Medium (6–7)	−0.15 (−0.71, 0.41)	0.10 (−0.36, 0.56)
High (≥8)	0.24 (−0.34, 0.83)	0.45 (−0.03, 0.93)
**Peer friendship characteristics**		
**Friends’ average physical activity**	0.20 (−0.31, 0.70)	0.61 (0.10, 1.13) *
**Popularity**	0.01 (−0.02, 0.04)	0.00 (−0.03, 0.02)
**Sent friendships**	0.03 (0.01, 0.05) *	0.02 (0.01, 0.04) *
**Constant**	7.28 (3.07, 11.49)	2.32 (−0.98, 5.62)
*R* ^2^	*0.25*	*0.20*

* *p* < 0.05 (two-tailed); Ref: Reference group. Fully-adjusted model included forced entry of all main effects.
